# Three-Axis Plate for Open Rigid Internal Fixation of Base Fracture of Mandibular Condyle

**DOI:** 10.3390/jfb16050186

**Published:** 2025-05-19

**Authors:** Marcin Kozakiewicz

**Affiliations:** Department of Maxillofacial Surgery, Medical University of Lodz, 113 Żeromskiego Str., 90-549 Lodz, Poland; marcin.kozakiewicz@umed.lodz.pl; Tel.: +48-42-6393422

**Keywords:** metallic biomaterial, plate, mandible, condyle, basal, fracture, treatment, open rigid internal fixation, processus condylaris mandibulae, osteosynthesis

## Abstract

Metallic biomaterials are prevalent in medical applications. In the treatment of mandibular fractures, the use of metallic biomaterials makes it possible to recover the ability to bite and partially recover speech through preventing ankylosis of the temporomandibular joints, the formation of pseudoarthritic joints, and the consolidation of reduced bones. This article presents the concept of a triaxial plate for osteosynthesis of basal fractures of the mandibular condyle, which are very common fractures in humans. Approximately half of patients with such fractures have wide (squat) condylar processes, which allows for the use of as many as three straight plates. However, installing three plates is quite troublesome, and the use of a single and transversely reinforced plate would facilitate treatment. This study proposes a plate with three reinforcements running along three divergent axes. The plate is fixed to the bone fragments with 11 screws. This concept for the treatment of basal fractures allows patients to quickly recover their primary system functions due to rigid fixation through the use of short (4 mm) screws, as there is no trauma to the medial pterygoid muscle and the mandible canal contents and no intermaxillary immobilization.

## 1. Introduction

Metals have been used as biomaterials for a long time [1,2,3,4,5,6]. In the early stages of development, sufficient strength and suitable mechanical properties were the main considerations for metal implants. With the development of new generations of biomaterials, biodegradable materials were proposed for treating mandible condyle fractures [7]. Biological functional design is very important for metal implants in biomedical applications [8]. In traumatology, biodegradable metals can enable the restoration of lost mandibular function. The most commonly used materials are biostable metals, which can help to restore occlusion and biting and chewing functions.

**Figure 1:**
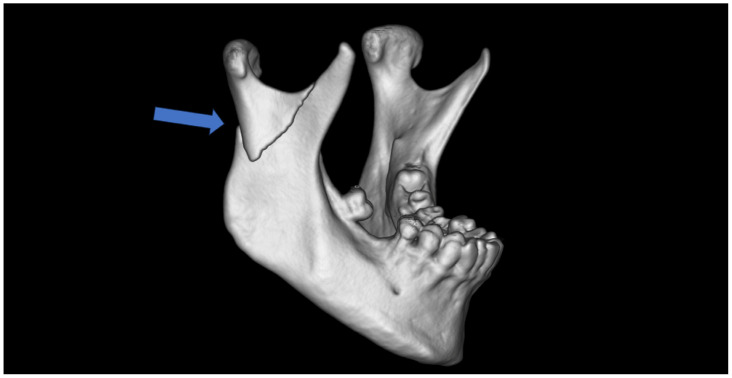
Typical base fracture of mandibular condyle (the arrow), in this case, on the right side. Lateral overlap and ramus shortening are also typical clinical observations.

The most common fracture of the condylar process of the mandible is a fracture at the base (Figure 1), which affects half of the patients with fractures of the condylar process. Fractures of the condylar process are also the most common mandibular injury. They can lead to malocclusion, affect biting and chewing, and can cause facial asymmetry due to lateral deviation of the chin [9,10]. Thus, effective treatment of condylar base fractures is a very important issue in maxillofacial traumatology [11,12,13,14,15,16,17,18,19,20].

Thanks to Christophe Meyer’s groundbreaking research, the stress lines of the bone in this anatomical region of the human skeleton are known [21,22,23,24]. Fractures of the base of the condylar process are concentrated in these stress areas. The first site is located along the posterior edge of the mandibular ramus and is in the area of natural bone augmentation, which is subjected to occlusal stresses (compressive forces act here). The mandible is thick here, allowing for the use of 8–9 mm long screws. The second biomechanically indicated site is just below the mandibular notch. Here, the bone is thin, and the forces acting on it are tensile. Fixation here is much more difficult, but is highly important. Due to the thinness of the bone, short screws are usually used here, as screws longer than 4 mm will perforate through the bone. This is mechanically ineffective and poses a risk of damaging the structures lying underneath the mandibular notch.

**Figure 2:**
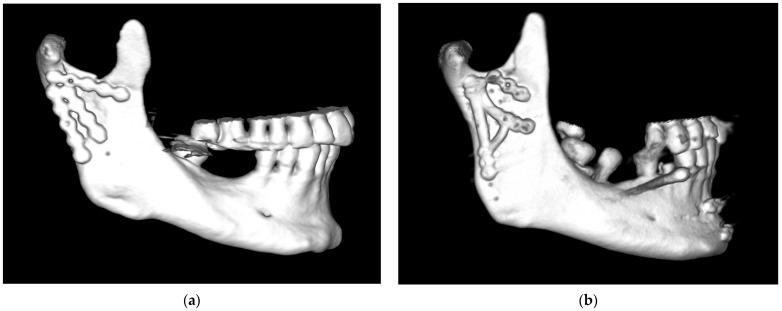
Examples of problematic osteosynthesis in basal fractures of squat condylar processes of the mandible: (**a**) Complicated basal fracture of the condylar process of the mandible. Fixation at the sites of ideal osteosynthesis (i.e., at the posterior edge of the condyle and under the mandibular notch), with the third plate located somewhere between the classic plate screwing sites. (**b**) Osteosynthesis with three straight plates that appear to be unstable (visible drill sites). Finally, the lower plates were replaced with a dedicated plate (but it is clearly too narrow for such a squat condylar process). Eventually, rigid fixation was achieved. Unfortunately, this unique strategy is not always successful.

This problem is particularly pronounced in patients with wide condylar processes and mandibular rami. While there is plenty of room for plate fixation, one of the biomechanically indicated osteosynthesis sites is still difficult to use (the mandible notch). Anatomical evaluations have indicated that people with wide condylar processes account for nearly half of the population. In such an anatomically wide, fractured condylar base, three plates or other non-typical combinations of dedicated plates with a straight plate can be used (Figure 2).

**Figure 3:**
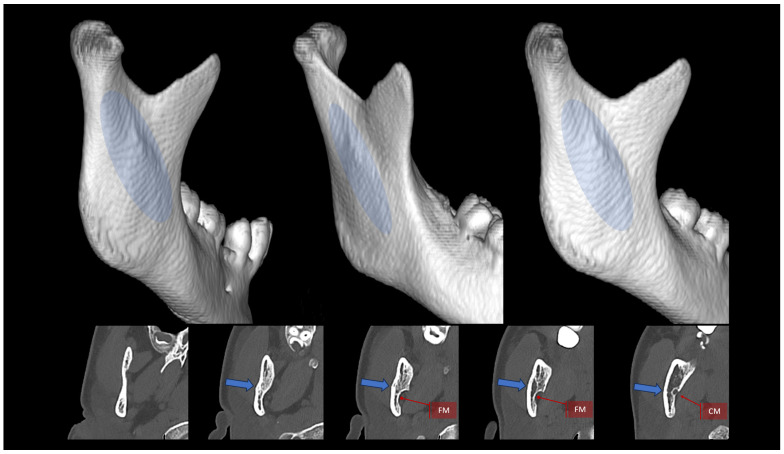
Appearance and location of the oblique augmentation of the condylar process and ramus of the mandible. Top: three-dimensional reconstructions created from a CT scan. Blue ellipse indicates the course of this lateral bony augmentation from the mandibular neck downward and anteriorly toward the ante-gonion notch. Bottom: horizontal cross sections. The blue arrow indicates the lateral augmentation. FM—foramen mandibulae; CM—canalis mandibulae. The radiological multiplanar reconstructions were performed using RadiAnt (www.radiantviewer.com/en; access date: 30 November 2024).

A third (additional) plate can be placed somewhere between the two ideal osteosynthesis sites mentioned earlier (Figure 2a). This takes advantage of the commonly occurring bone thickening that runs obliquely from the condyle downward and anteriorly (Figure 3). Finding all the bone fragments, and reducing and fixing them anatomically with such a set of plates, requires considerable dexterity on the part of the surgeon. It would be much easier to use a single plate that already has three plates oriented in different directions.

Here, the author proposes a new dedicated plate for functional osteosynthesis of fractures at the base of the condylar process of the mandible.

## 2. Materials and Methods

### 2.1. Design

**Figure 4:**
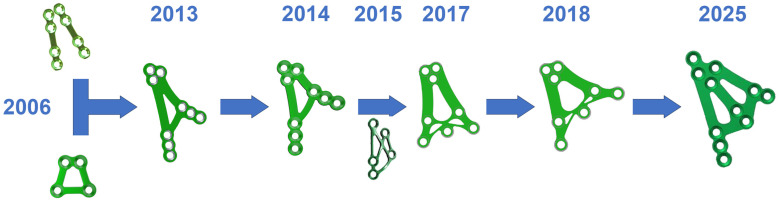
Development of a plate material design for a wide bone base over a span of several years. The idea of a 3AXP plate for osteosynthesis at the base of the mandibular condylar process has been taking shape over the last 10 years.

The development of a plate for a wide bone base is presented in Figure 4. Many years ago, the biomechanical advantages of fixing two simple plates were combined with the versatility and transverse reinforcements of a dedicated “small” delta plate (e.g., from Medartis; www.medartis.com; access date: 6 May 2025). Next, attempts were made to improve the biomechanical properties by changing the arrangement of holes in the lower part of the plate. A hole between the lower ends of the lower arms was added. This was the first time oblique anatomical augmentation of the condylar process was considered using dedicated plates. Then, such a plate with X-shaped struts was adapted for the broad treatment of fractures at the base of the condylar process. This strut construction resulted from the analysis of stresses in these strut plates (from Synthes; www.jnjmedtech.com/en-US/companies; access date: 6 May 2025). The results for the strut plate were surprisingly good. Ultimately, however, the strut structure of the plates was abandoned, which was preceded by mechanical tests of several dozen different types of plates and the indication that wide, longitudinal reinforcements produce the best biomechanical effects for condyle osteosynthesis.

A three-axis plate was proposed as a solution to the abovementioned problem. The main design idea is to add a third arm to ACP- or XCP-type plates, which would run along the lateral anatomical augmentation at the condylar process and mandibular branch (Figure 3). Thus, this three-axis condylar plate (3AXP) has 3 strong arms connected transversely at the bottom by crossbars, which contain 4 holes for fixing using System 2.0 screws at the top. In the lower part, each arm would end with two or three holes. There are 11 holes for screws in total throughout the 3AXP.

### 2.2. Assessment

Preliminary finite element analysis (FEA) was performed to compare osteosynthesis using classical straight plate versus 3AXP fixation of a base fracture of the mandible condyle. For the boundary conditions, the incisor teeth in the model were fixed in three directions but could rotate, and the condyle could translate on the plane surface of a support. The loads on the incisor used reflect a mouth opening of 5 mm, which is the condition that causes the most tension and displacement in the condyle. The muscular actions applied here are similar to the ones presented in the maxillofacial literature, and five principal muscles were included in the loading configuration: the deep masseter (X = 7.776 N, Y = 127.23 N, Z = 22.68 N), superficial masseter (X = 12.873 N, Y = 183.5 N, Z = 12.11 N), medial pterygoid (X = 140.38 N, Y = 237.8 N, Z = −77.3 N), temporalis (X = 0.064 N, Y = 0.37 N, Z = −0.13 N), and medial temporal (X = 0.97 N, Y = 5.68 N, Z = −7.44 N) muscles. The FEA used in the present study was composed of 1 million tetrahedral linear elements. The FEA was performed using SolidWorks software 2025 (www.solidworks.pl accessed on 10 May 2025). The simulations took into account the mechanical properties of mandible cortical bone: a Young’s modulus of 14.7 GPa and a Poisson coefficient of 0.48. The Young’s modulus and Poisson coefficient of the implant and screws were assumed to be 113.8 GPa and 0.32, respectively. The implants were applied to the right side of the mandible in the first model, with 3 straight plates used as a reference, and in the second model, the 3AXP was used. The implants fixed the fracture at the mandible condyle base. In simulating the behavior of the screws, they were considered to be completely surrounded by cortical bone. The screw–implant contact was modeled as a touching contact situation. No contact was modeled between the implant and bone (0.3 mm separation).

### 2.3. Implant Specifications

**Figure 5:**
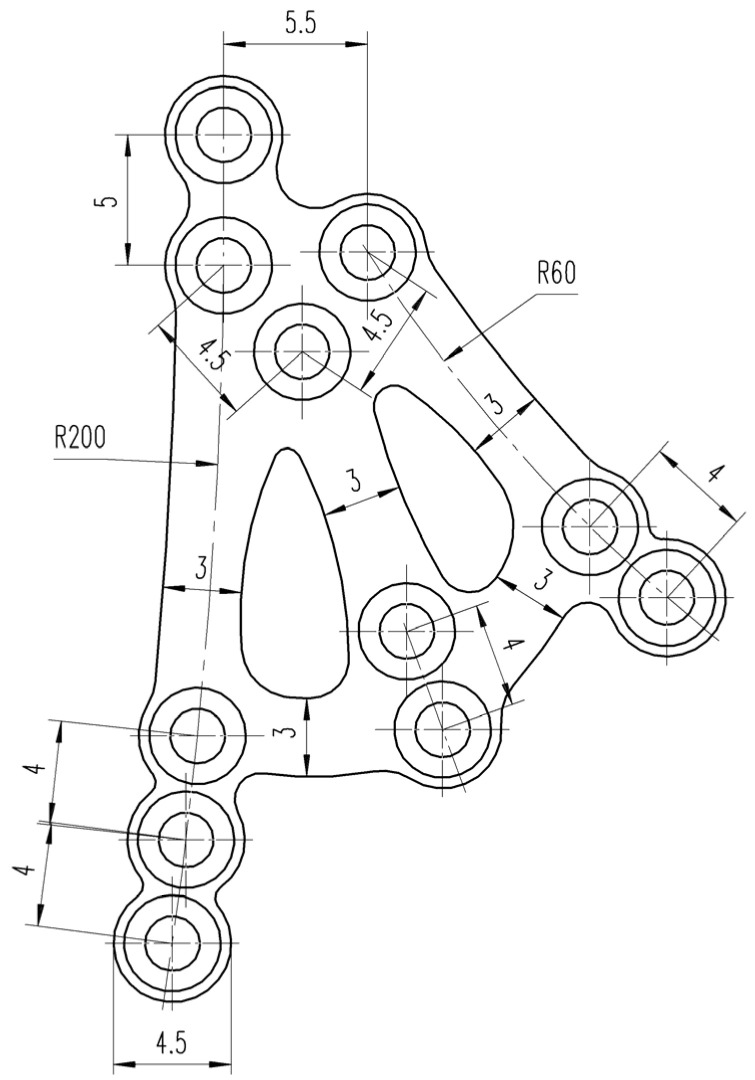
Technical drawing of the 3AXP.

The final implementation of the above idea is this plate (Figure 5) connecting reinforcements running along three divergent axes. This is a rather large design, but it still falls into the category of mini-plates (System 2.0) rather than reconstruction plates (Systems 2.4–2.8). From the top group of four holes, reinforcement arms (bridges) that are 3 mm in width run along the three axes. The upper and middle arms are finished with a group of two holes for 4 mm long screws. The third arm, the posterior arm, is terminated by a series of three holes. All the holes in the plate are chamfered. The lower ends of the arms with holes are connected by 3 mm wide crossbars.

The material used for the implant was annealed grade 2 titanium. According to the ISO 5832-2 standard, the chemical composition of the implant is shown in Table 1. The plate material was characterized in accordance with the above standard and had the following mechanical properties: tensile strength Rm = 345 MPa, yield strength Rp0.2 = 275 MPa, elongation after fracture A = 20%, and reduction of area Z = 30%.

### 2.4. Manufacturing

The process of manufacturing the 3AXP consisted of the following stages: abrasive waterjet cutting from the titanium sheet, machining (milling, polishing, washing and drying), anodizing the surfaces, washing and drying, heat treatment, laser marking, a final washing, packing and labeling, and a final control step.

**Figure 6:**
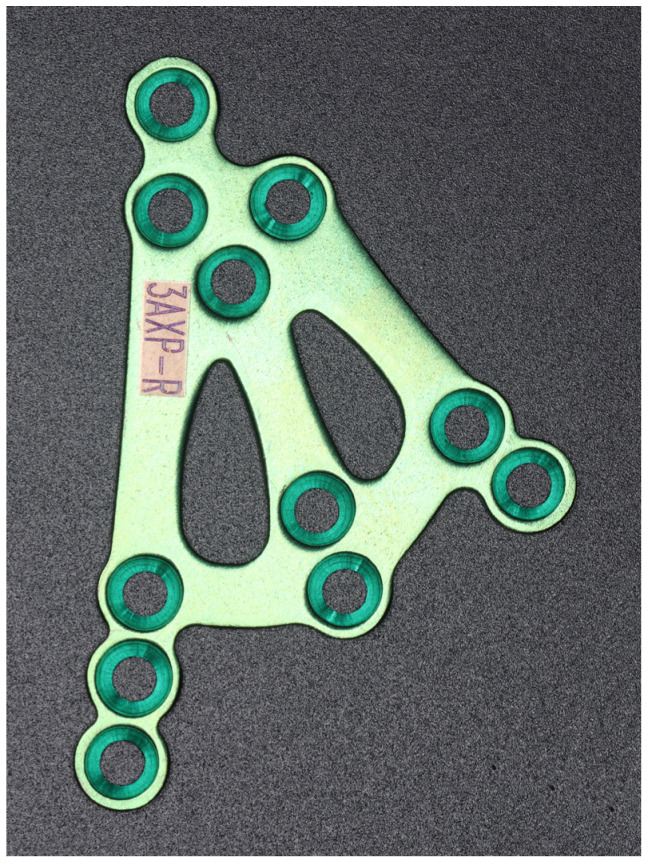
Image of the 3AXP (three-axis condylar plate) for the right side. The plate thickness is 1.2 mm, the arms and cross-bars are 3 mm in width, and the diameter of the holes is 2.0 mm.

The plate was cut from a 1.2 mm thick sheet. The final height of the 3AXP was 35.5 mm with a width of 23 mm. The holes for the screws were chamfered to ensure that most of the occlusal forces were transmitted through the bone and not through the plate. The plate was designed to induce load-sharing osteosynthesis to reduce the risk of plate fracture (Figure 6). For these reasons, the plate is dedicated to each side of the patient separately.

## 3. Results

**Figure 7:**
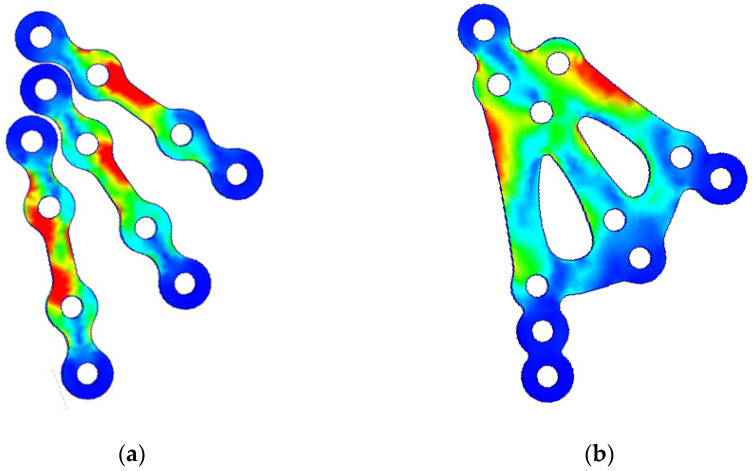
Comparison of stress on straight mini-plates versus 3AXP in osteosynthesis of the same fracture at the base of the mandibular condyle. (**a**) Stress in three-plate fixation system. (**b**) Stress in three-axis plate. Stress concentrations (red areas) at the edges of the holes indicate the places where the fixation material is most prone to fracture. In the 3AXP, these areas of stress concentration are some distance from the holes.

A preliminary numerical evaluation of the stress occurring in the 3AXP was compared to that of typical fixation using straight plates (in this case, the closest reference system was three-plate osteosynthesis). After occlusal loading on the three-plate fixation system, stresses appeared in each plate near the middle bridges. These were concentrated at the second hole from the top in each plate. In the plate fixed closest to the posterior edge of the condyle, stress concentrations also occurred at the third hole from the top. In the 3AXP system, the stresses were farther from the edges of the holes and were concentrated at the solid top of the plate. In both osteosynthetic materials, the peripheral holes (the top-most and the bottom-most holes) were subjected to low levels of stress (Figure 7).

**Figure 8:**
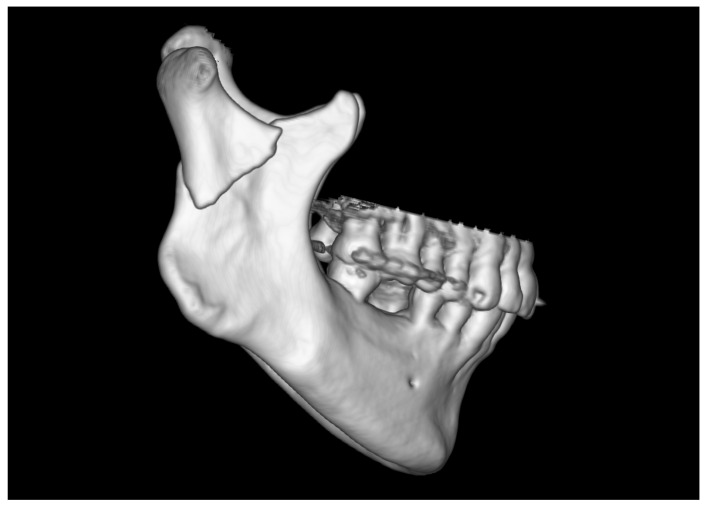
Radiological image from a multi-row CT scan. The examination was performed on the day of admission to the hospital. A fracture of the base of the right condylar process of the mandible with displacement (i.e., overlap of fragments in fracture site) was observed.

**Figure 9:**
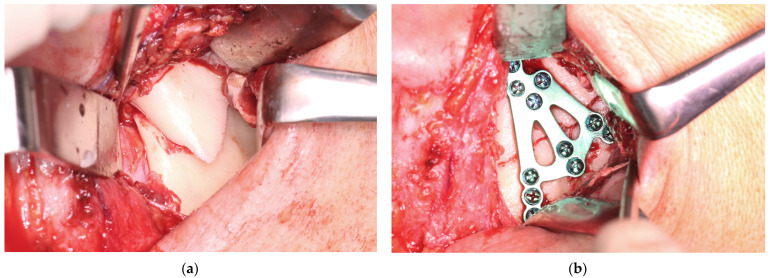
Clinical application of the 3AXP. (**a**) Fracture in the base of the mandible condyle (note the typical lateral overlap of the bone fragments). (**b**) Open reduction and rigid fixation using triaxial plate. The used screws were a 2.0 mm system.

The clinical result of a 3AXP application (ref. 3.7321.0000; manufacturer: ChM; www.chm.eu; access date: 23 April 2025) is presented in Figure 8 and Figure 9. The patient (male, 42 years old) reported in person to the emergency room immediately after being hit by another player’s elbow during a basketball game. The victim did not lose consciousness. It was a direct injury to the masseter region on the right side. The patient noticed a tilt in the bite on the side of the injury at the last molar and the bite opening in the anterior segment (Helkimo Index was 3). There were no additional bone fractures in either the craniofacial region or the rest of the skeleton. An enamel fracture was noted in the upper right canine tooth (class II according to Ellis and Davey). The facial muscle function was not affected (based on the House–Brachmann scale, the function was scored as a 1).

On the second day after the injury, he underwent general anesthesia with nasal intubation. Maxillomandibular immobilization is not used in such fractures as an adjunct to open treatment. The retromandibular surgical approach was extended slightly to reach the lower part of the preauricular one (the skin incision line reached the lower pole of the tragus). In the first part of the procedure, the superficial musculo-aponeurotic system of the face (SMAS) was reached and followed anteriorly. Approximately 1 cm anterior to the posterior edge of the mandible, the SMAS was transected vertically along with the parotideo-masseterica fascia to the masseter. The marginal branch of the mandible (moved downward) and the buccal branch of the facial nerve (moved upward) were located. The masseter was cut vertically. Two bone fragments located in the displaced position were exposed (Figure 9a).

**Figure 10:**
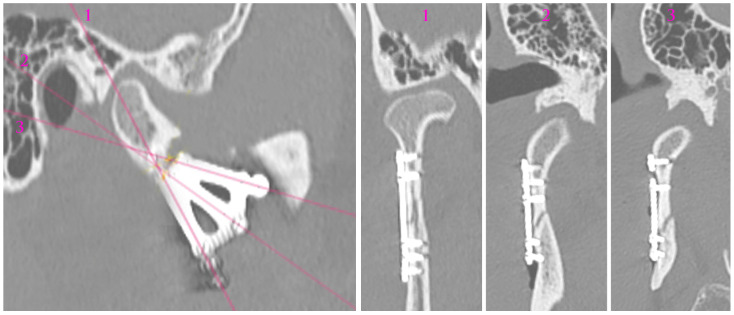
Radiological visualization of post-operational localization of the plate. The three cross sections (1, 2, 3) of different planes show the screwed plate at the osteosynthesis site. In cross-sections 2 and 3, the lower screws (four in total) are 4 mm long. The other screws are 6 mm long. Note that none of the screws point towards either the foramen or the mandibular canal.

**Figure 11:**
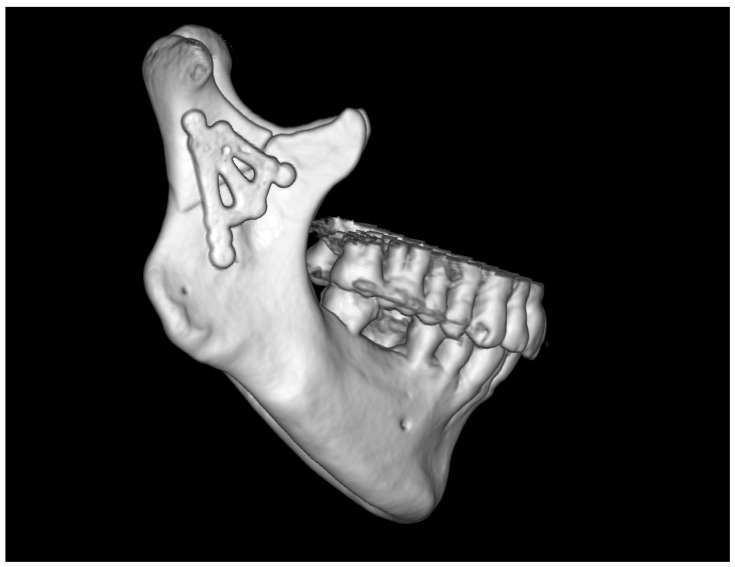
Three-dimensional reconstruction image of treatment results from CT. The bone fragments are anatomically reduced. Occlusion was stabilized by the 11 screws fixing the plate.

The skeletal traction was then anchored in the right mandibular angle, and wire was brought through the skin to the outside and fastened to the instruments for improved grip. After the administration of a muscle relaxant, the bones were reduced into the anatomical position. The 3AXP was inserted onto the bone surface and screwed to the proximal fragment by self-tapping with a 6 mm long screw. First, one screw was inserted, and then the 3AXP was positioned and fixed with one screw in the distal fragment. Then, the remaining three screws were inserted into the proximal fragment and tightened firmly. To make the mandibular notch visible, two 4 mm long screws were inserted into the distal fragment. Then, another two 4 mm long screws were inserted in the lower part of the middle arm of the plate. Intraoperative tomography was performed to check the position of the screws in the thin bone. At this stage of the procedure, all that remained was to add the last two screws (6 mm long) in the lower part of the plate along the posterior condylar edge. The quality of the reduction and narrowness of the fracture gap was evaluated to complete the internal reduction and rigid fixation of the condylar base fracture (see Supplementary Materials). At this stage, the skeletal traction was removed. The result of the radiological examination is shown in Figure 10 and Figure 11.

No complications were noted in the patient’s facial nerve function (see Supplementary Materials). The patient was discharged on the third day after the surgery without maxillomandibular immobility and with a recommendation of a liquid diet for 7 days and then a soft diet for 5 weeks. After this time, no occlusal disorders were noted (Angle class I, the same as before the injury); the mouth opening was recorded at 50 mm, the laterotrusion to the left was 8 mm, and the laterotrusion to the right was 14 mm. The Helkimo Index value was 0; according to the House–Brachmann scale, the facial muscle function was scored a 1. The patient returned to their professional activity on the 8th day after discharge from the hospital.

**Figure 12:**
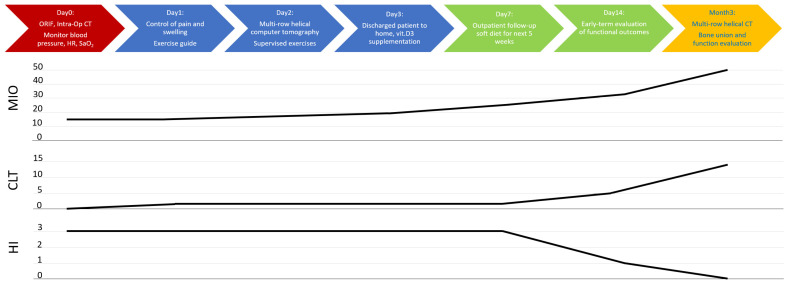
Summary of applied clinical protocol and timeline of clinical recovery. Abbreviations: ORIF—open rigid internal fixation; Intra-Op—intra-operational; CT—computed tomography; HR—heart rate; SaO_2_—blood oxygen saturation level; vit. D3—vitamin D3; MIO—maximal interincisal opening [mm]; CLT—contralaterotrusion [mm]; HI—Helkimo Index.

The typical protocol adopted for the treatment of simple mandibular fractures for patients in good general condition was followed for this patient (shown in Figure 12). It did not need to be modified at all, either for medical reasons or for patient cooperation.

**Figure 13:**
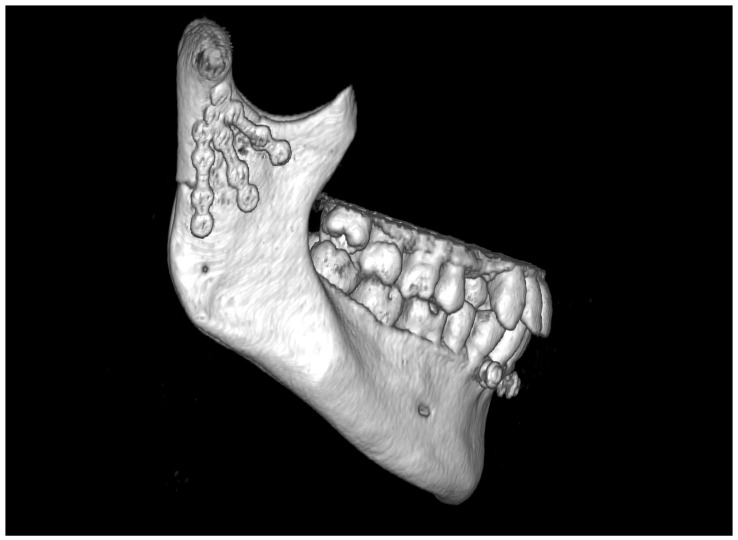
Treatment results of application of three straight plates. Note that there are no transverse connectors between the plates. The crucial difference compared to the use of the 3AXP is the large mass of metal attached to the proximal portion of the mandible. In the 3AXP, there are 4 screws clustered in the upper portion, while the image above has 6.

**Figure 14:**
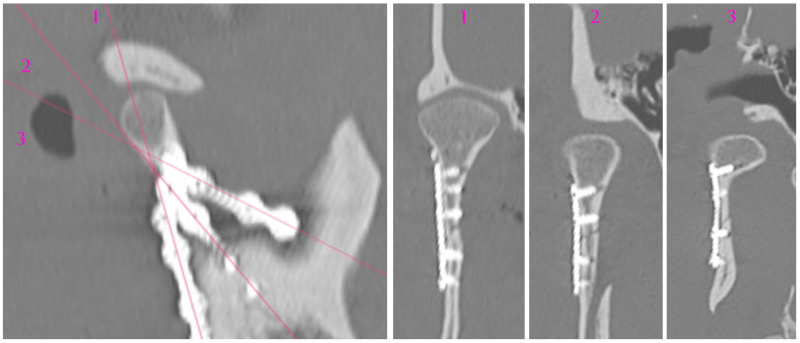
Analysis of screw location in plates The three cross sections (1, 2, 3) of different planes show the screwed plate at the osteosynthesis site. It is still possible to select screws of different lengths to avoid drilling through the bone, but the lack of transverse connectors between the plates does not reduce the risk of the plate loosening.

An alternative method for inducing osteosynthesis for this type of fracture is using a third reinforcement in the anatomical augmentation region. This can be achieved using standard straight plates (Figure 13 and Figure 14). Ideally, the plates should have a reinforced bridge to prevent plate fracturing. This was based on the numerical experiment presented at the beginning of Section 3.

When using three straight plates, one has to find room for 12 screws in the mandibular condylar process. Although this is possible, handling three plates is much more difficult than using one dedicated plate (the 3AXP also requires fewer screws). Obviously, the fixation will have sufficient stability, but prolonged maneuvering during open reduction and plate screwing will cause greater trauma to the masseter, retromandibular vein, superficial temporal artery, and nerve trunks. For the patient, a shorter surgical procedure makes a difference.

## 4. Discussion

The functional metallic materials used for internal bone fixation can be divided into two types: load-bearing and load-sharing [25,26,27]. They are used as part of the standard treatment of trauma for all parts of the skeleton. 

Load-bearing materials take on all stresses induced by vital functions. In the case of mandible and condylar process fractures, these functions are biting and chewing food. The forces are significantly high and can lead to a fracture of the plate. This often occurs at a hole in the plate, which is why the arms in dedicated plates are designed without holes (including the 3AXP). Usually, a load occurs when the fragments of the condylar process are insufficiently reduced and the patient does not follow medical advice. To prevent this, the patient could not use their mandible for a long period of time (maxillomandibular immobilization for 6 weeks), but this is very inconvenient for patients and questionably effective in noncompliant patients.

The load-sharing of metallic materials is achieved by creating bevels in the plate around the screw holes. Such chamfered holes allow the plate to be pressed against the surface of the fixated bone fragments. This type of hole is used in the 3AXP. It is assumed that part of the occlusal load will be transmitted by the interlocked bone fragments. Only the remaining part of the load will be carried by the plate’s metal alloy material. This integration of the bone structure with the function of the plate helps support bone union.

The 3AXP is designed for load-sharing, with reinforcement from its material (thickness increased to 1.2 mm titanium alloy) and structure (three wide bridges without holes).

**Figure 15:**
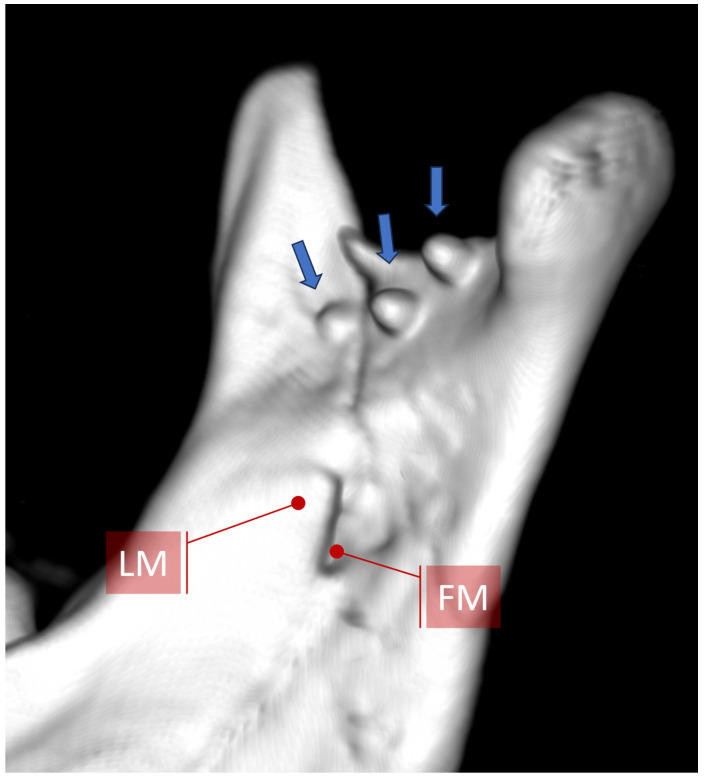
View of the medial surface of the mandibular condylar process after osteosynthesis of the basal condylar fracture using 6 mm long screws. When screwing the plate below the mandibular notch, it should be remembered that the use of screws longer than 4 mm is usually pointless, since they pierce through the bone into the pterygo-mandibular space (arrows) and do not increase the rigidity of fixation. If 6–8 mm long screws are inserted opposite the mandibular foramen or canal, the neurovascular bundle could be damaged (compare with Figure 16). Abbreviations: LM—lingula mandibulae; FM—foramen mandibulae.

**Figure 16:**
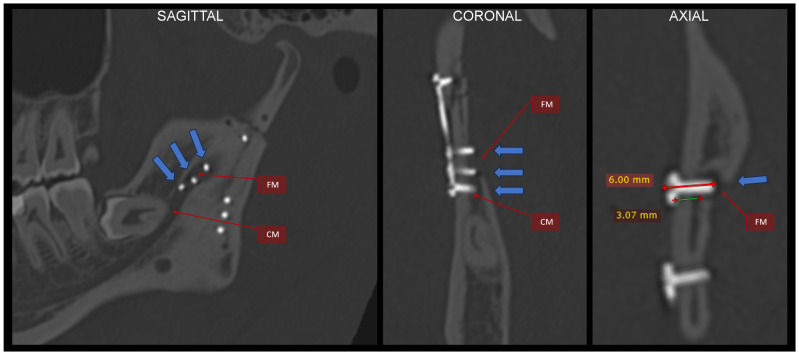
Justification for why short screws should be used. An example of using a plate with two lower divergent arms. In each of these arms in the lower part, there are 3 holes for screws. Three screws located in the middle of the mandibular ramus puncture through the bone and penetrate the mandibular foramen and the initial part of the mandibular canal (see arrows). This position of the implants was revealed by an intraoperative CT scan and was quickly corrected. The blue arrows indicate this medial bone perforation. It is worth noting that in this case, the bone separating the mandibular canal from the outer surface of the ramus is approx. 3 mm. If one adds the thickness of the plate (1.0–1.3 mm), then the screws that are 4 mm in length will not reach the mandibular canal. The radiological multiplanar reconstructions were performed using RadiAnt (www.radiantviewer.com/en; access date: 30 November 2024). Abbreviations: FM—foramen mandibulae; CM—canalis mandibulae.

The objective of the surgical treatment of fractures is anatomical reduction and rigid fixation. Despite some researchers’ belief that some mobility is needed in the fracture gap during healing [28,29,30,31,32,33,34], in the case of osteosynthesis of mandibular condylar process fractures, it does not even manage to come close to pre-traumatic stability. Therefore, it is necessary to direct all stabilization efforts to achieve the most rigid internal fixation of the condylar process. This is achieved using screws inserted into the bone through the holes in the plates. There are two options for the type of screw: self-tapping and self-drilling screws. If one can afford higher pressure during osteosynthesis, self-drilling screws provide higher stability at the same length as a self-tapping screw. A second advantage is that they can avoid pre-drilling, which can sometimes lead to the perforation of thin bone (Figure 15 and Figure 16).

It is also known that a longer screw requires a higher force to pull it out. Here, however, one must keep in mind anatomical limitations. In particular, in this part of skeleton, the mandibular canal and the location of the mandibular foramen are crucial. The foramen upper edge is 12 mm below the mandibular notch, and its posterior edge is 4 mm from the posterior edge of the condylar process. In each patient, the treatment is planned using a CT scan, and it is necessary to pay attention to the displacement of the fragments as well as where the fixation screws will be located. The ideal lines of condylar osteosynthesis avoid the mandibular foramen and canal because they lie at the posterior condylar edge just below the mandibular notch [21]. This is very convenient. However, the thin bone at the notch makes fixation difficult. Moreover, in this region, osteosynthesis is often complicated by an intermediate bone fragment, and this area could end up not being very stably fixed. This is problematic since tensile-tearing forces are exerted here during biting and chewing [21,35], and it is only possible to insert 4 mm long screws here. This situation is somewhat improved by the possibility of using 4–5 screws on both sides of the fracture line if one can find enough room for so many screws. A triaxial plate could solve this problem.

A triaxial plate has a typical upper arm and a posterior arm whose position after fixation should be close to the course of the ideal osteosynthesis lines [21]. Due to the thin bone and the course of the maxillary artery near the mandibular notch, the upper arm should be fixed here with 4 mm long screws (Figure 10 and Figure 14). The posterior arm runs along the posterior edge of the condyle, so 6 mm or even 9 mm long screws can be used. Of course, in the upper part of the plate (proximal bone fragment), the holes should be fixed with 6 mm long screws.

There is also an intermediate arm on the distal fragment that provides support for the poor rigidity of the osteosynthesis below the mandibular notch. This arm sometimes lies on the oblique line of mandibular augmentation. Again, 4 mm long screws should be used here because these points are already above the mandibular canal in patients with condylar base fractures (Figure 10 and Figure 11). Thus, four 4 mm long screws supported by three screws at least 6 mm in length are placed in areas of the reduced distal bone fragment. The total number of screws fixing the plate is as many as 11 since the number of screws also determines the stability of the fixation.

The plate was designed for the half of the population with squat condylar processes [21] and a base fracture of the mandibular condylar process. This plate is not suitable for fractures of the condylar process neck and may not be suitable for patients with a slender condylar process. For these fractures and people, there are many other dedicated plates, or two straight plates can be used.

**Figure 17:**
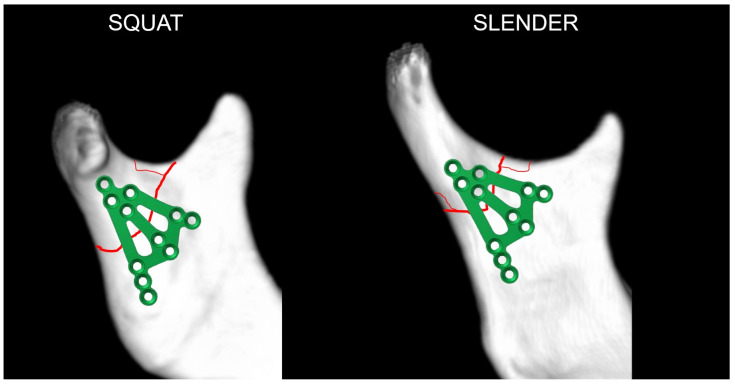
The triaxial plate can be applied in most cases of a condylar base fracture independent of the anatomic variant of the mandible. This expands the group of patients who can benefit from its use. It is worth recalling here that the ratio of a squat to a slender process is 47 to 53, so the 3AXP could be used in most patients with condylar base fractures.

Finally, the author concluded that the anatomical type of mandibular condylar process does not matter when applying a new plate. In slender condyles, it is also possible to apply 3AXP for many fractures at the base of the condyle (Figure 17). Thus, the most important indication for treatment with the triaxial plate is a fracture of the condylar base running through a wide space of bone regardless of the anatomical variant. How many such fractures occur in slender condyles remains a question for epidemiologists.

Therefore, in a medical center treating condylar process fractures, there should be plates available for high-neck (narrow and high) and low-neck (e.g., some kind of “delta” plate) mandibular fixation. The mentioned plates can be successfully used in osteosynthesis of condylar base fractures in slender anatomical variants. It is useful to have a triaxial plate for condylar base fractures in squat condylar processes as well as for a part of slender condylar processes. The versatility and good rigidity of osteosynthesis of using two straight plates is also advantageous, and they can produce good functional results and are very economical. As always, the surgeon is the most important factor, and the surrounding implants are a secondary matter. The use of the 3AXP forces the fracture fixation to follow known ideal osteosynthesis lines [21,22], even when the surgeon is unaware of them: when one tries to place the plate on the bone surface, the 3AXP width causes the plate arms to align along the stress lines in the condylar process.

The advantages and disadvantages of the presented osteosynthesis plate are listed in Table 2.

Further material modifications such as using magnesium alloy biodegradable plates [26] or surface conditioning [36] to reduce ion release from the titanium alloy could be considered [37,38,39].

Since some fractures of the base of the condylar process cannot be properly treated in a closed fashion (e.g., dislocated [40,41]), it may be interesting to cite some results of closed and open treatments [42]. It is worth emphasizing that closed treatment fails to anatomically reduce the fragments, there is a large disproportion in the height of the ramus, and very common results include malocclusion, muscle pain, and arthropathy (Table 3).

One may say that the choice of treatment method is simply a consideration of the clinical trade-offs of treatment (e.g., maxillomandibular immobilization). However, the author believes that this is not a compromise, but the responsibility of the doctor. The research [42] indicates that denying a patient open treatment (when it is known that it would cure them) could condemns them to craniomandibular dysfunction or temporomandibular disorders. Thus, the choice of treatment method must not be based on the doctor’s predilection or fears, or even “avoidance medicine”, but on the objective indications for treatment.

Generally, maxillofacial traumatology is a persistent challenge in healthcare systems. There is an ongoing transition in maxillofacial traumatology treatment from younger to older patients who may be more infirm and thus more vulnerable to the effects of injury, highlighting the increasing importance of interdisciplinary treatment of patients with pre-existing conditions in maxillofacial surgery [43]. The implementation of injury prevention measures might be beneficial in this population, despite having the best conceptualized tile designs and strategies for their use.

## 5. Conclusions

The presented plate concept may prove useful in osteosynthesis of basal fractures of the mandibular condylar process in a significant number of patients who have a squat condyle. These patients can be expected to benefit in terms of a rapid return of stomatognathic function.

## Figures and Tables

**Table 1:** The chemical composition of the grade 2 titanium used for the three-axis plate.

Element	Maximum Compositional Limit (% Mass Fraction)
Nitrogen	0.03
Carbon	0.08
Hydrogen	0.012
Iron	0.30
Oxygen	0.25
Titanium	Balance

**Table 2:** Summary of the clinical benefits and limitations of the 3AXP.

Benefits	Limitations
1. Allows stable fixation	1. Large dimensions
2. Solves the problem of using 2 short screws in a biomechanically important location (under the mandibular notch)	2. Not suitable for the treatment of fractures of the neck or all basal fractures in the “slender” anatomical variant
3. Helps treat fractures of the base of the condylar process in the “squat” anatomical variant of the mandible 4. Protects the condyle and the pterygoid-mandibular space from being drilled through 5. No need for maxillo-mandibular immobilization 6. Quick stomatognathic function recovery	3. Material is not resorbable 4. Requires a surgical procedure 5. Need for studies on a large number of patients

**Table 3:** Comparison of treatment outcomes of open versus closed treatment [41].

Feature	Open	Closed
No complaints	74% of patients	27% of patients
Muscle pain	13% of patients	38% of patients
Arthropathy	13% of patients	62% of patients
Maximal interincisal opening	>45 mm	>45 mm
Contralaterotrusion	9 mm	7 mm
Loss of ramus height	0.77 ± 0.88 mm	5.40 ± 3.21mm
Anatomic reduction of proximal fragment	Yes	No
No restoration of occlusal conditions	26% of patients	46% of patients

## Data Availability

The original data presented in the study are openly available in YouTube at https://www.youtube.com/@marcinkozakiewicz5618 (accessed on 17 May 2025). The raw data supporting the conclusions of this article will be made available by the authors on request at www.researchgate.net/profile/Marcin-Kozakiewicz (accessed on 1 April 2025).

## Supplementary Materials

The following supporting information can be downloaded at: https://www.mdpi.com/article/10.3390/jfb16050186/s1, Video S1: Reduction; Video S2: Reduction; Video S3: Fixation; Video S4: Fixation; Video S5: Occlusion and facial nerves.
